# Breaking barriers: Aptamers in ocular disease treatment

**DOI:** 10.1016/j.omtn.2024.102399

**Published:** 2024-12-02

**Authors:** Tao Wang, Rakesh Naduvile Veedu

**Affiliations:** 1 The Kids Research Institute Australia, The University of Western Australia, Nedlands, WA 6009, Australia; 2 Centre for Molecular Medicine and Innovative Therapeutics, Health Futures Institute, Murdoch University, Murdoch, WA 6150, Australia

## Main text

Ocular diseases, including visual impairment and blindness, are projected to affect over 280 million people by 2040, imposing substantial economic burdens and drastically reducing the quality of life for those affected.^1^ Due to the existence of a blood-retinal barrier, current treatments often involve invasive procedures, such as intravitreal injections, which lead to serious side effects and poor patient compliance. There is a pressing need for more efficient and patient-friendly treatment options.

The study by Sonja Korhonen et al. introduces a novel approach using *in vivo* SELEX (systematic evolution of ligands by exponential enrichment) to select RNA aptamers capable of penetrating ocular barriers after intravenous administration.^2^ This approach could revolutionize ocular pharmacotherapy by offering a more efficient and patient-friendly treatment option.

Aptamers are short single-stranded RNA or DNA molecules that can adopt three-dimensional shapes in solution. Due to their excellent target binding affinity and specificity, low-cost production, and potential for chemical modifications to enhance stability, aptamers hold great potential in drug development.^3^

In fact, applying aptamers to treat ocular diseases is not a new idea. In 2004, a 28-mer chemically modified RNA aptamer targeting vascular endothelial growth factor (VEGF) protein called pegaptanib (Macugen) was approved by the FDA for the treatment of wet age-related macular degeneration (AMD).^4^ However, the invasive intravitreal administration and unsatisfactory treatment effect greatly compromised its broad applications compared with the antibody drug alternatives. These limitations might be due to its *in vitro* selection procedure. Although the identified aptamers demonstrated desired targeting affinity in the *in vitro* selection system, there were concerns about their ability to effectively penetrate ocular tissues and recognize their targets inside the body, which has very different conditions from the *in vitro* system. Considering this limitation, the *in vivo* SELEX performed by Sonja Korhonen and colleagues is of great significance. Performing the selection procedure in the body rather than in buffering systems enables the development of ocular-barrier-penetrating aptamers capable of recognizing targets in physiological conditions.

In this study, the authors utilized intravenous injection in rats, followed by extracting and identifying aptamers directly from various ocular and control tissues, including the conjunctiva, cornea, iris-ciliary body, lens, retina, and retinal pigment epithelium-choroid. The researchers conducted 14 *in vivo* SELEX cycles, and next-generation sequencing (NGS) was used to analyze the aptamer pools from SELEX cycles 10, 12, and 14, yielding a total of 14,482 unique sequences. The study focused on sequences with high abundance, identifying five different aptamer families based on nucleotide similarity.

Three lead aptamers—Apt1, Apt2, and Apt5—were eventually identified based on their specific accumulation in vascularized ocular tissues. Apt2 and Apt5 showed significant differences in biodistribution between ocular and control tissues, indicating their potential to be systematically administered in ocular disease treatment, offering a less invasive systemic administration and more patient-friendly alternative to current methods. Interestingly, Apt1, despite being the most abundant in the sequencing pool, did not show a significant difference in biodistribution, raising questions about the factors influencing aptamer efficacy, such as the incubation scheme or PCR bias.

In summary, the innovative approach reported in the study sets the foundation and offers great promise for future research into the use of aptamers in ocular pharmacotherapy. The ability to target specific ocular tissues with high precision opens up possibilities for treating a wide range of ocular conditions, including AMD, diabetic retinopathy, and glaucoma. Notably, although this study demonstrated great promise, further research is needed to assess the long-term safety and efficacy of these aptamers in human subjects, given the different physiological settings and protein expression patterns between humans and rats. Future research should focus on validating these findings in human subjects and exploring the broader applications of aptamers in molecular and cellular therapies. Additionally, exploring the mechanisms underlying aptamer targeting and biodistribution could provide valuable insights for optimizing their therapeutic potential.

## Declaration of interests

The authors declare no competing interests.
